# Addressing current challenges in optimization of lipid management following an ACS event: Outcomes of the ACS EuroPath III initiative

**DOI:** 10.1002/clc.23988

**Published:** 2023-02-17

**Authors:** Alberico L. Catapano, Raffaele De Caterina, J. Wouter Jukema, Robert Klempfner, Ulf Landmesser, François Schiele, Alessandro Sionis

**Affiliations:** ^1^ Department of Pharmacological and Biomolecular Sciences University of Milan Milan Italy; ^2^ Division of Cardiovascular, Department of Cardiothoracic and Vascular, Pisa University Hospital, University of Pisa, Pisa, Azienda Ospedaliero‐Universitaria Pisana, Pisa, and Fondazione Villaserena per la Ricerca Città Sant'Angelo Pescara Italy; ^3^ Department of Cardiology Leiden University Medical Center Leiden The Netherlands; ^4^ Netherlands Heart Institute Utrecht The Netherlands; ^5^ Cardiac Rehabilitation Institute, Sheba Medical Center Tel‐Aviv University Tel Aviv Israel; ^6^ Department of Cardiology, Charité University Medicine Berlin, German Centre for Cardiovascular Research (DZHK), Partner Site Berlin Berlin Institute of Health (BIH) Berlin Germany; ^7^ Department of Cardiology, University of Burgundy Franche‐Comte University Hospital Jean Minjoz Besançon France; ^8^ Department of Cardiology, Hospital de la Santa Creu i Sant Pau, IIB‐Sant Pau Universitat Autònoma de Barcelona Barcelona Spain; ^9^ Centro de Investigación Biomédica en Red de Enfermedades Cardiovasculares (CIBERCV) Madrid Spain

**Keywords:** acute coronary syndromes, cardiovascular risk, LDL cholesterol, lipid‐lowering treatments, myocardial infarction, PCSK9 inhibitors

## Abstract

**Background:**

Low‐density lipoprotein cholesterol (LDL‐C) lowering is key to reduce atherosclerotic disease progression and recurrent events for patients after acute coronary syndrome (ACS). However, LDL‐C management for post‐ACS patients remains challenging in clinical practice.

**Hypothesis:**

The ACS EuroPath III project was designed to optimize LDL‐C management in post‐ACS patients by promoting guideline implementation and translating existing evidence into effective actions.

**Methods:**

Three surveys targeting cardiologists (*n* = 555), general practitioners (GPs; *n* = 445), and patients (*n* = 662) were conducted in Europe, with the aim of capturing information on patient characteristics and treatment during acute phase, discharge and follow‐up. GPs’ and patients’ opinions on key treatment aspects were also collected. Based on survey results, international experts and clinicians identified areas of improvement and generated prototype solutions. Participants voted to select the most feasible and replicable proposals for co‐development and implementation.

**Results:**

Five key areas of improvement were identified: (1) inappropriate treatment prescribed at discharge; (2) lack of lipid guidance in the discharge letter; (3) inadequate lipid‐lowering therapy (LLT) optimization; (4) gaps in guideline knowledge and lack of referral practices for GPs; (5) patients’ concerns about lipid management. Proposed solutions for these focus areas included development of a treatment algorithm for the acute phase, a standardized GP discharge letter, an assessment tool for LLT efficacy at follow‐up, an education plan for GPs/patients and a patient engagement discharge kit. The standardized GP discharge letter and treatment algorithm have been selected as the highest priority solutions for development.

**Conclusion:**

These initiatives have the potential to improve adherence to guidelines and patient management after ACS.

## Graphical abstract

The ACS EuroPath III project was designed to optimize lipid management in post‐ACS patients. Following data collection through 3 surveys, 5 key areas for improvement were identified including inappropriate treatment prescribed at discharge, lack of lipid guidance in the discharge letter, inadequate LLT optimization, gaps in guideline knowledge and lack of referral practices for GPs, and patients’ concerns about lipid management. Solutions were proposed for each of these issues, with the generation of a treatment algorithm and a standardized patient discharge letter prioritized for early development.

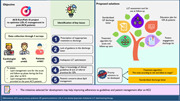

## 1 INTRODUCTION

Cardiovascular disease (CVD) is the commonest cause of death in Europe, causing over 4 million deaths annually.^1^ Acute coronary syndromes (ACS), including unstable angina and myocardial infarction (MI), are life‐threatening manifestations of CVD.^2^, ^3^ Approaches in the acute‐phase management of ACS (including interventional and pharmacological treatment) have evolved over time, and post‐MI mortality has fallen, but could still be reduced further.^4^, ^5^

Management of low‐density lipoprotein cholesterol (LDL‐C) level is one of the least controlled modifiable risk factors in post‐ACS patients.^6^, ^7^, ^8^, ^9^ Current guidelines recommend new LDL‐C reduction goals for patients post‐ACS.^6^ To this end, these goals were lowered from 1.8 mmol/L (70 mg/dL) to 1.4 mmol/L (55 mg/dL) for all patients with ACS, with the additional aim of achieving at least 50% reduction from baseline; an even lower goal of 1 mmol/L (40 mg/dL) is recommended for patients with recurrent events.^6^ It is now important to ensure the implementation of current guidelines into clinical practice, so as to reduce the risk of negative outcomes in patients post‐ACS.^7^

Based on this background, the ACS EuroPath project (initiatives I–III) was designed to help optimize post‐ACS lipid management and improve clinical outcomes. Details of the ACS EuroPath I and II forums have been published previously^10^, ^11^; a brief overview can be found in Section S1. The aim of the ACS EuroPath III initiative (2020–2021) was to translate existing evidence and guidelines on key topics into accessible, effective, and well‐structured solutions, to address identified gaps and optimize post‐ACS lipid management in clinical practice. Here we describe the proposed solutions, filtered through a pre‐ordered process involving European cardiologists, general practitioners (GPs) and patients.

## 2 METHODS

The ACS EuroPath I survey was conducted in 2018, with 555 cardiologists participating.^10^ Two further surveys were conducted by Ipsos MORI in 2020, targeting GPs and patients. These surveys were completed by 445 GPs providing data on 1780 patient cases, and 662 patients. These surveys captured information on patient characteristics and treatment during acute phase, at discharge and during follow‐up; GPs and patients also provided feedback on their own knowledge base, confidence level regarding treatment and what they considered to be key aspects of treatment. Full methodological details are provided in Section S2.

The ACS EuroPath III initiative aimed at identifying and selecting five focus areas for possible improvement, based on data from the specifically designed surveys described briefly above. Members of the ACS EuroPath III forum worked together via a digital ideation platform and a series of “hackathon” (hacker marathon) remote meetings to research, develop and define appropriate solutions for each key area. Solutions were intended to be universally applicable, regardless of factors relating to regional practice or local healthcare systems. A total of 42 international experts from 18 countries engaged in the ACS EuroPath III initiative (full details in Section S3), and the five final solutions were pitched to participants during the third and final hackathon meeting, after which participants voted on solutions to prioritize for detailed development and implementation.

## 3 RESULTS

### 3.1 Key findings from the surveys

#### 3.1.1 ACS EuroPath I (i.e., cardiology) survey

Results from the Cardiology survey have been reported in full previously.^10^ In brief, 555 physicians participated and the study enrolled 2775 patients with ACS. For patients in acute phase, 91% of patients were tested for lipid levels, mostly (73%) within the first day of hospitalization. During hospitalization, 93% received an LLT; of them, 37% were already under LLT and 56% were statin‐naïve patients. Of patients who received statins, the majority (64%) received them on day 1 of hospitalization; however, the average was 2.3 days. At discharge, only 66% of the patients received a high‐intensity statin (HIS) therapy. For follow‐up, few (14%) patients had a follow‐up within 4–6 weeks of discharge, with 3 months as an average timeframe. The majority of patients (77.6%) at the first follow‐up had LDL‐C > 70 mg/dL; of them, 41% had no change to their LLT. Similar data were observed during the second follow‐up visit.

#### 3.1.2 GP survey

Key demographics are shown in Table 1A. A total of 445 GPs participated in the survey, contributing data from 1757 patients. Average length of hospitalization was 11 days. Slightly less than half (46%) of patients were newly initiated on LLT at discharge from hospital (typically an HIS monotherapy), while 36% who were already on LLT maintained their therapy with adjustments. The first follow‐up consultation after discharge was equally likely to be with a GP or a cardiologist; the majority (43%) had their first follow‐up with a GP within 6 weeks of discharge. Nearly half (47%) of the patients had lipid levels 71–100 mg/dL at follow‐up, and 27% of patients had lipid levels at 55–70 mg/dL. Over half (58%) of patients were currently receiving an HIS, with only 3% receiving a PCSK9 inhibitor. Combination therapy was prescribed to less than 10% of patients. Overall, 68% of patients had their LLT reassessed across all follow‐ups; the main reason GPs gave for reassessing LLT was to check patient compliance (54%) rather than maximize LDL‐C reduction (42%). Overall, changes were made to patient LLT in 27.3% of cases across all follow‐ups.

**Table 1:** Key demographics for the (A) GP and (B) patient surveys.

A	
GP survey	
Total number of GPs included	445
France	103
UK	117
Spain	117
Italy	108
Average number of years in practice	17
Secondary specialties (number of GPs)	
Internal medicine	45
Cardiology	41
Diabetology	34
Other/no second specialty	325
Average 3‐month caseload of patients with ACS (number of patients)	32
Total number of patient records included	1757
Average age, years	66
Sex, male	74%
Comorbidities	
Hypertension	73%
Obesity	47%
Type 2 diabetes	42%
Family history of CVD	41%
Experiencing first ACS event	87%

B	
Patient survey	
Total number of patients included	662
France	143
UK	150
Spain	123
Italy	150
Netherlands	48
Sweden	48
Average age, years	57
Sex, male	76%
Comorbidities	
High cholesterol	39%
High blood pressure/hypertension	32%
Overweight	26%
Diabetes	21%
Family history of CVD	59%
Admission for most recent heart attack	100%
Timing of most recent heart attack	
6–9 months previously	40%
10–13 months previously	34%
14–18 months previously	26%

Abbreviations: ACS, acute coronary syndrome; CVD, cardiovascular disease; GP, general practitioner.

Most (94%) GPs of the survey indicated that they believe managing lipid levels is very important; however, a lower proportion (66%) felt very confident in their ability to manage them. The main barriers to optimal lipid management for patients with ACS (from a GP perspective) were identified to be patient lack of understanding (58%), patient nonadherence (56%) and GPs’ inability to prescribe PCSK9 inhibitors (42%). Most (94%) GPs discuss lipid levels with their patients during consultations (always, 53%; regularly, 41%). Lipid management information is received by 73% of GPs, generally embedded with other information. More than half (60%) of GPs surveyed are aware of clinical guidelines for managing ACS patient lipid level, with most (~70%) feeling confident in implementing the ESC/EAS recommendations. The main reason given for nonimplementation (by 48% of respondents) is that GPs are unable to initiate/prescribe PCSK9 inhibitors.

#### 3.1.3 Patient survey

Key demographics are shown in Table 1B. Overall, 662 patients participated. The majority (90%) of patients stated the belief that managing cholesterol levels is important to prevent another CV event. During hospitalization, high cholesterol is the main CV risk factor discussed with healthcare professionals (HCPs). Most (90%) patients discussed cholesterol management with cardiologists during hospitalization and most (89%) were satisfied with the discussion. Half were taking a statin before their most recent event, while at discharge this proportion increased to over 60%. Approximately one‐third of the patients received no prior treatment before their most recent ACS event. Patients spent a mean of 6.3 nights in hospital, and approximately half of respondents (46%) experienced only positive emotions at discharge (20% experienced only negative emotions). Discharge letters were received by most (94%) patients’ GPs, with the majority (46%–48%) including information on LLT prescribed, cholesterol levels, and timings for next consultation. Most (84%) patients were satisfied with the follow‐up care after their most recent heart attack. A total of 61% of patients had their first follow‐up within 6 weeks of discharge, with the next 2–3 follow‐up consultations generally falling within 3 months of discharge. Patients were most likely to see a cardiologist at their first 3 follow‐up consultations (53%, 48%, and 40% of patients at the first, second and third consultations, respectively), and a cardiac rehabilitation doctor thereafter (48%, 54%, and 66% of patients at the fourth, fifth and sixth consultations, respectively). Rehabilitation programs were planned at discharge for 70% of patients. Most programs were planned to start within 6 weeks of discharge, lasting on average 10 weeks.

#### 3.1.4 The ACS EuroPath III initiative

The five key focus areas for ACS EuroPath III initiative were: (1) inappropriate treatment prescribed at discharge; (2) lack of lipid management guidance in the discharge letter; (3) inadequate LLT optimization; (4) gaps in knowledge of clinical guidelines and lack of referral practices for GPs; and (5) patients’ concerns about blood lipid management (Table 2). Following completion of the ideation phase, five solutions (one to address each key area of unmet need highlighted by the surveys) were proposed, as follows:

**Table 2:** Key areas for improvement; concerns supporting selection of these areas.

Key areas for improvement	Concerns supporting this selection
(1) Inappropriate treatment prescribed at discharge	Lack of lipid blood test in some patients during acute phase Delayed introduction of statins Lack of LLT prescribed at discharge after an ACS event in some patients Consequences of inappropriate treatment at discharge (i.e., patients not at LDL‐C goal) Suboptimal LLT intensification before discharge
(2) Lack of guidance in the discharge letter	Late communication/delivery of discharge letter to GPs Lack of consistent lipid management guidance in discharge letter Inconsistent communication of discharge letter information to patient
(3) Inadequate LLT optimization	Late 1st follow‐up (>6 weeks) for majority of patients LDL‐C levels above goal at 1st follow‐up Suboptimal LLT treatment Inconsistent LDL‐C assessment at 1st follow‐up
(4) Gaps in knowledge of clinical guidelines & lack of referral practices for GP	Lack of GP awareness of clinical guidelines Lack of knowledge regarding optimal lipid treatment management Lack of GP comfort in prescribing nonstatin therapies Lack of GP comfort in referral (to another GP or to specialist)
(5) Patient's concerns about blood lipids management	Lack of patient knowledge regarding cholesterol level management Patient nonadherence to LLT Lack of patient education on lipid management

Abbreviations: ACS, acute coronary syndrome; LDL‐C, low‐density lipoprotein C; LLT, lipid‐lowering therapy; GP, general practitioner.

### 3.2 Inappropriate treatment prescription at discharge

#### 3.2.1 Survey findings

Key issues identified in the Cardiologist survey included the lack of lipid blood test within 1 day of admission and high number of patients who received statin therapy on day 2 or later. In addition to this, approximately one‐third of patients were not receiving HIS at discharge or had LDL‐C > 70 mg/dL at the first follow‐up; data from the GP survey support the last two points. Current guidelines recommend that HIS should be initiated as soon as possible following an ACS event,^4^, ^5^ and that a lipid profile should be obtained after 4–6 weeks postdischarge to determine if the therapeutic goal has been reached. If not, optimization of the LLT with ezetimibe and/or PCSK9 inhibitor is needed.^4^, ^6^ Viewing these survey data in the light of current guideline recommendations, action is needed to address inappropriate treatment prescription at discharge.

#### 3.2.2 Proposed solution

We propose the development of an algorithm for use by hospital cardiologists during the acute phase to clarify the LDL‐C goal and the recommended LLT treatment approach (based on the “*Fire early according to risk and follow to goal/target*” approach). This encompasses lifestyle, HIS, ezetimibe, and PCSK9 inhibitors, and should involve a systematic follow‐up visit (by a cardiologist or a GP) at 4–6 weeks after introduction of the LLT to determine the clinical and biological tolerance and efficacy. We recommend that physicians should take full advantage of recent openings in permission to prescribe PCSK9 inhibitors even in the hospitalization phase in various European countries.

#### 3.2.3 Key comments/takeaways

Using a dedicated algorithm, treatment with HIS plus ezetimibe should be initiated as soon as possible following an ACS event in all patients. Selected patients may require additional LLT (i.e., a PCSK9 inhibitor) during hospitalization, as per 2019 ESC/EAS recommendation.^6^ At follow‐up, the threshold for adding PCSK9 inhibitors can be determined based on various criteria: (1) according to ESC guidelines, when LDL‐C remains above the therapeutic LDL‐C goal (>1.4 mmol/L [55 mg/dL]); (2) according to the design of the clinical studies with PCSK9 inhibitors, when LDL‐C is >1.8 mmol/L (70 mg/dL); or (3) according to local rules, because the conditions of availability and reimbursement vary across European countries (Section S4). If needed, PCSK9 inhibitors should be considered and introduced early after ACS based on the findings of the ODYSSEY‐OUTCOMES study. Patients should be monitored closely by the physician responsible for their follow‐up care (i.e., specialist or GP) after being discharged, to assess LLT tolerance, adherence, effectiveness, and whether further LLT intensification is required. To this end, a 4–6‐week follow‐up visit is recommended; this makes it possible to initiate management of statin‐associated muscular symptoms if needed and enables LLT optimization. Cardiologist follow‐up should be considered for selected patients.

### 3.3 Lack of lipid management guidance in the discharge letter

#### 3.3.1 Survey findings

In the GP survey, the majority (90%) of respondents confirmed that their patients received discharge letters, usually within 7 days. Overall, 64% of respondents reported that lipid management guidance was included in the discharge letter; this information included details of specific LDL‐C goal, when the next measurement of LDL‐C levels should be carried out, lipid treatment guidance and appropriate timing for follow‐up visits. Interestingly, 41% of respondents reported that the lack of lipid management information in the discharge letter was a barrier to optimal lipid management. Respondents recommended that inclusion of information on specific LDL‐C goal, lipid treatment algorithm, timing of next LDL‐C measurement, and optimal hospital follow‐up time in the discharge letter would help them to optimally manage patients with ACS.

#### 3.3.2 Proposed solution

Clear rules on how to prepare an optimal discharge letter are needed. This may take the form of a template of a standardized discharge letter for GPs, focusing on the individualized status and risk of the patient, the importance of treating to goal, and clear recommendations for LLT and follow‐ups. A list of proposed strategies for addressing LLT approaches in a discharge letter is provided in Table 3.

**Table 3:** Strategies for addressing lipid lowering treatment approaches in a patient's discharge letter.

Use clear and concise language to make the document readable for general practitioners and patients
Include a short summary, either at the beginning or at the end of the document, stating the cause of the admission, the management strategy, and the individual risk of the patient
Provide information regarding LDL‐C levels at admission and previous LLT treatment
State individualized LDL‐C goals (both mg/dL and mmol/L) and a percentage of LDL‐C reduction from baseline (e.g., your desired LDL‐C goal is 1.4 mmol/L)
Give precise instructions that are easy for patients to follow (e.g., name of the drug, dose, posology, route of administration and duration of treatment)
Stress the importance of reaching the goals as soon as possible (e.g., according to current scientific evidence, the LDL‐C goal should be achieved as soon as possible in order to reduce the patient's residual cardiovascular risk)
Stress the importance of treatment adherence (e.g., the effectiveness of the treatment is highly dependent on good adherence and will offer lifelong protection)
Mention the timing (ideally between 4 and 6 weeks after discharge) and place of the planned follow‐up visits and blood analysis
Offer a referral pathway for patients that do not reach the desired cardiovascular risk factors goals or experience drug‐related adverse events (e.g., statin‐related muscle symptoms)
Be proactive and, whenever possible, engage your patient in a structured cardiac rehabilitation program
Suggest trusted sources of information that are easily accessible for your patient, as the information received by patients during the admission is usually very fragile

Abbreviations: LDL‐C, low‐density lipoprotein C; LLT, lipid‐lowering therapy.

#### 3.3.3 Key comments/takeaways

GPs receiving a standardized discharge letter with key information relating to lipid management will benefit from clear, individualized guidance on each patient's risk status and recommended LLT. Such a standardized discharge letter might help all the clinicians involved in the post‐ACS patient management. Patients will benefit from an efficient, standardized, and time‐sensitive approach to treatment, which may improve adherence and achievement of LDL‐C goals to reduce recurrence of cardiovascular events.

### 3.4 Inadequate LLT optimization

#### 3.4.1 Survey findings

Data from the Cardiologist survey indicate that almost half of patients who had not achieved LDL‐C target at first follow‐up had no subsequent change made to their LLT treatment regimen. In the GP survey, respondents noted that their main aim in reassessing LLT was to check patient compliance, and changes were made to patient LLT in approximately one‐quarter of cases. Almost half of all patients whose data were included in the GP survey saw a GP at the first follow‐up. The fact that follow‐up may be conducted by one of many different HCPs, in diverse settings, makes it hard to ensure LLT is being thoroughly assessed, especially since one‐third of GPs expressed a lack of confidence in their own ability to manage patient lipid levels. In addition to this, timing of the first follow‐up visit was variable; guidelines suggest that patients should be seen for follow‐up 4–6 weeks after an ACS event,^6^ but average time from discharge to first follow‐up in the Cardiologist survey was 3 months, with few patients meeting the timeframe suggested in guidelines. These data suggest that there is a need to improve the process by which LLT is optimized.

#### 3.4.2 Proposed solution

A structured process enabling the systematic assessment of the efficacy of LLT at the 4–6‐week follow‐up, regardless of the setting in which it is conducted. Telemedicine appointments are a feasible alternative and could be used to evaluate the results during follow‐up.^12^ At this stage, the results are shared and the next steps for optimal management are discussed.

#### 3.4.3 Key comments/takeaways

All patients should be discharged on high‐intensity LLT. Follow‐up blood test and visits should be scheduled according to a predefined process (e.g., regular intervals between visits, with specified times at which particular blood tests should be carried out), and the patient should be engaged in the follow‐up process. It should be noted that for most patients, the maximum level of LLT will be the maximum tolerated statin dose, plus ezetimibe. In patients at very high CV risk who are predicted not to reach target LDL‐C levels, the option also exists to add PCSK9 inhibitor treatment at discharge.^6^ If access to follow‐up appointment with cardiologists is limited, visits should be scheduled with alternative HCPs according to patient risk and predicted need for ongoing adjustments to LLT.

### 3.5 Gaps in knowledge of clinical guidelines and lack of referral practices for GPs

#### 3.5.1 Survey findings

GPs face a major challenge when striving to keep up to date with current guidelines in all fields of medicine and may have limited time for research and consultation. As such, data from the GP survey indicate that 40% of respondents are not familiar with the 2019 ESC/EAS clinical guidelines.

#### 3.5.2 Proposed solution

A three‐step plan to educate GPs and patients to update them with current guidelines, the safety and efficacy of LLT, and generate a call‐to‐action to ensure effective post‐ACS lipid management:

‒ Awareness campaign by well‐known GPs, cardiologists and influencers on the goals and importance of post‐ACS lipid management.
‒ Clearer protocols that enable GPs to assess goal attainment and refer patients when needed.
‒ Emphasizing of safety and substantial benefit of LLT in achieving guideline goals and engaging both patients and GPs as guardians of the evidence‐based lipid goals.

#### 3.5.3 Key comments/takeaways

Priming GPs on the need for change, ensuring that patients and GPs are on the same page with regard to the importance and key elements of their care, and using credible sources (e.g., ESC, EAS and national cardiac society websites) to reinforce how treatment is strictly linked to patient outcomes, will have a positive impact on GPs' workload and patient outcomes. Establishing simple referral criteria for lipid management consultations is likely to improve consultation rates, as well as easing LLT management modifications. To this end, it would be worthwhile to consider ways to remove any existing bureaucratic barriers. Referral for consultation from within the medical record could potentially reduce the amount of time physicians spend entering redundant information into numerous forms, and the use of well‐designed asynchronous tele‐consultation platforms would also be of benefit. Further to this, tools that have the capacity to integrate with a patient's electronic medical record could be used to highlight lipid values above goal and help provide reminders for laboratory retesting, thus improving standard–ization of care.

### 3.6 Patient concerns about blood lipid management

#### 3.6.1 Survey findings

Over the last two decades there has been a progressive shortening of the length of hospital admissions in patients with ACS.^4^, ^13^ Current guidelines recommend an early discharge (<48–72 h in low‐risk patients after an ACS), although the data on hospital stay from the GP and Patient surveys (ranging from 6 to 11 days) show that in practice patients may remain for longer. Whatever their length of stay, patients may be fatigued/overwhelmed and emotionally variable, with around one‐third of patients experiencing a mixture of positive and negative emotions. It is reasonable to suggest that many patients may be less able to absorb necessary information and key messages about their ongoing treatment on their own at the point of discharge, and that some assistance might be usefully supplied.

#### 3.6.2 Proposed solution

Develop a “patient engagement discharge kit” designed to be offered to patients during their discharge briefing. The “discharge kit,” should include concise written and visual instructions summarizing the key information, the importance of following instructions, as well as links to trusted websites for additional supporting information. This will enable HCPs to deliver the following key messages: reasons why the patient experienced an ACS, what steps they need to follow going forward, and potential consequences if they do not follow these steps (i.e., Why did you have an ACS? What happens now? What will you have to do to reduce the risk of a recurrent event? What happens if you do not do it?). The discharge instructions for the patient should ideally include a recommended LDL‐C goal.

#### 3.6.3 Key comments/takeaways

Having access to a patient engagement discharge kit will enable cardiologists and other HCPs (e.g., nurses) to provide patients with a package of clear, simple instructions on next steps, thus potentially benefitting both parties. If possible, it would be good to link the patient discharge kit to a digital platform (e.g., CardioSmart, LipidApp) offering addition information/detail as needed.

#### 3.6.4 Ranking and prioritization of pitched solutions

Following discussion, the hackathon participants voted for their preferred pitch solutions, based on their potential to improve post‐ACS lipid management and their applicability to clinical practice. Participants ranked the patient engagement discharge kit and the treatment algorithm as being most likely to improve lipid management, and the patient engagement discharge kit and standardized GP discharge letter as being most easily implemented in clinical practice. The participants voted to prioritize the standardized GP discharge letter and treatment algorithm for development and implementation.

## 4 DISCUSSION

In the ACS EuroPath III forum, participants agreed that implementation of a Patient engagement discharge kit and treatment algorithm would have the greatest impact on clinical outcomes, but decided to prioritize the standardized GP discharge letter and treatment algorithm for initial development and implementation. Evidence from a recent review of eight European initiatives to optimize post‐ACS lipid management suggests that the application of treatment algorithms, standardized identification of high‐risk patients, routine screening for familial hypercholesterolemia and intensification of LLT can significantly increase the achievement of LDL‐C goals, as well as decrease CV risk of recurrent events.^7^ The here‐described forum decided to identify the five most relevant possible areas of intervention and to rank them in terms of priority, feasibility and importance.

The use of GP discharge letters has previously been investigated, most notably in the Discharge Communication Study; as part of this study, factors influencing the perceived usefulness of a latter were identified.^14^ Key “useful” factors included reason for admission, patient diagnosis, a list of procedures performed and test/examination results. It was noted that the use of clear language in discharge letters is essential for safe and effective transition of care, and that such letters should be concise and understandable (for both GP and patient).^14^ Therefore, development of a standardized discharge letter taking into account previously‐identified “useful” elements would be logical. One such standardized letter template has previously been suggested in a position paper endorsed by ILEP and includes suggestions relating to timings of GP/cardiologist follow‐up, advice on ongoing treatment/monitoring and next steps if LDL‐C goals are not met.^15^ In the opinion of authors, the concept of a standardized GP discharge letter for patients post‐ACS is worth developing further, ideally at a pan‐European level.

The use of treatment algorithms in healthcare is widespread, with different clinical areas developing approaches as needed^16^, ^17^; it has been suggested that an ideal treatment algorithm should be explainable, dynamic, precise, autonomous, fair and reproducible.^18^ The ACS EuroPath III forum suggests that the widespread uptake and utilization of a treatment algorithm for the acute‐phase ACS management would be beneficial in terms of patient outcomes and also aid hospital cardiologists in making effective clinical decisions at an early stage. Ideally, such an algorithm should be locally adapted to accommodate for the different reimbursement policies in European countries.

The ACS EuroPath III initiative identified key unmet needs in post‐ACS lipid management according to the feedback from both physicians (cardiologists and GPs) and patients. The survey results, together with those of ACS EuroPath I, provided a relatively comprehensive view of the gaps in care between clinical practice and guidelines from both the physicians’ and patients’ perspectives. Solutions were developed by a broad group of 42 experts from 18 European countries, which will greatly benefit GPs in implementing lipid management guidelines in their daily practice. However, it should be of note that regional differences may lead to difficulties in implementation of chosen solutions and adjustments according to regional variation may be required. In addition, the possibility of selection bias (inherent to all observational studies) should be considered.

## 5 CONCLUSIONS

In the authors’ opinion, all the solutions proposed here would have a positive impact on clinical outcomes and will help to improve optimal lipid management for both HCPs and patients post‐ACS. It is hoped that there will be widespread uptake of the proposed standardized discharge letter and treatment algorithm, and that improvements in lipid management will become evident for patients post‐ACS. Future steps might be to monitor changes in practice, as suggested by these considerations, and their effects on measurable outcomes, such as the achievement of LLT goals and the time to achieve them from patients’ discharge after an ACS.

## AUTHOR CONTRIBUTIONS

All authors equally involved in study concept and design, acquisition of data, analysis and interpretation of data, drafting of the manuscript and critical revision of the manuscript for important intellectual content. All authors had access to relevant data, and participated in the writing, review, and approval of the manuscript.

## CONFLICTS OF INTEREST STATEMENT

Alberico L. Catapano: has received honoraria, lecture fees, or research grants from: Aegerion, Amgen, Amryt, AstraZeneca, Bayer, Daiichi‐Sankyo, Eli Lilly, Genzyme, Ionis Pharmaceutical, Kowa, Mediolanum, Medscape, Menarini, Merck, Mylan, Novartis, PeerVoice, Pfizer, Recordati, Regeneron, Sanofi, Sigma‐Tau, and The Corpus. The work of ALC is supported in part by Ministero della Salute Ricerca corrente.

Raffaele De Caterina: reports grants, personal fees and nonfinancial support from Sanofi during the conduct of this study; personal fees from Boehringer Ingelheim, Bayer, BMS/Pfizer, Daiichi‐Sankyo, Milestone, Novartis, Menarini, Guidotti, Sanofi and Roche, all outside the submitted work.

J. Wouter Jukema: his department has received research grants from and/or was speaker (with or without lecture fees) on a.o. (CME accredited) meetings sponsored by Amarin, Amgen, Athera, Biotronik, Boston Scientific, Dalcor, Daiichi‐Sankyo, Lilly, Medtronic, Merck‐Schering‐Plough, Novartis, Novo Nordisk, Pfizer, Roche, Sanofi Aventis, the Netherlands Heart Foundation, CardioVascular Research the Netherlands (CVON), the Netherlands Heart Institute and the European Community Framework KP7 Program.

Robert Klempfner: gave sponsored lectures and/or received consultation fees from Sanofi and AstraZeneca.

Ulf Landmesser: reports research grants to the institution from Amgen, Bayer and Novartis. Speaker or advisory honorary from Amgen, Daiichi‐Sankyo, Novartis, Sanofi.

François Schiele: reports personal fees from Amgen, AstraZeneca, Bayer, BMS, MSD, Pfizer, Lilly, Novartis, Mylan, Sanofi and Servier.

Alessandro Sionis: reports speaker fees and nonfinancial support from Amgen, AstraZeneca, Bayer, Boehringer Ingelheim, Daiichi‐Sankyo, Ferrer, Novartis, Sanofi and Servier; research grants from: AstraZeneca, Daiichi‐Sankyo, Ferrer, GE Health Care and Zoll.

## Supporting information

Supporting information.

## Data Availability

Qualified researchers may request access to patient level data and related study documents including the clinical study report, study protocol with any amendments, blank case report form, statistical analysis plan, and dataset specifications. Patient level data will be anonymised and study documents will be redacted to protect the privacy of our trial participants. Further details on Sanofi's data sharing criteria, eligible studies, and process for requesting access can be found at https://www.vivli.org/.
