# Supplementary material for: Addressing current challenges in optimization of lipid management following an ACS event: Outcomes of the ACS EuroPath III initiative
Source: Clin Cardiol. 2023 Feb 17;46(4):407–15. doi: 10.1002/clc.23988 (PMC10106658; doi:10.1002/clc.23988)
Supplement: Supplementary file 1 — Supporting information. [file CLC-46-407-s001.docx]

# Addressing current challenges in optimisation of lipid management following an ACS event: Outcomes of the ACS EuroPath III initiative

Alberico L. Catapano MD, PhD, Raffaele De Caterina MD, PhD, J. Wouter Jukema MD, PhD, Robert Klempfner MD, Ulf Landmesser MD, PhD, François Schiele MD, PhD, Alessandro Sionis MD, PhD

## ***Running Title***: ACS EuroPath III initiative: optimising lipid management

# Supplementary Materials

## Section 1: Brief overview of ACS EuroPath I and II surveys

## In the ACS EuroPath I project, a cardiologist survey was designed by the ACS EuroPath I steering committee (SC) to assess lipid management care for the acute and follow-up phase during the first year after an ACS.^1^ The survey was circulated to cardiologists in seven European countries and completed by 555 respondents, providing data on 2775 patients who had experienced an ACS event. Results of the survey were reviewed and discussed by the ACS EuroPath I team of 43 cardiologists, and key gaps in care between clinical practice and guidelines were identified.

## For the ACS EuroPath II forum (2019), 50 cardiologists discussed gaps in the post-ACS lipid management pathway versus care recommended in the ESC/EAS Guidelines.^2^ A healthcare professional (HCP) self-assessment tool was developed to help physicians evaluate their clinical practice versus guidelines.

## Section 2: Cardiologist, GP, and Patient surveys – methodology

### Survey methods

In ACS EuroPath I, the survey for cardiologists was conducted in 2018 in seven European countries, including France, Germany, Italy, Spain, United Kingdom, Switzerland and the Netherlands. Eligible participants were interventional or general cardiologists who had 3–35 years of practice with >70% of time spent in direct patient care and treated >20 patients with ACS per month. The main survey was administered as a 45-minute online questionnaire that included a patient record of the last five patients with ACS (acute or follow-up phase).^2^

For ACS EuroPath III, the GP survey was performed in four European countries, including France, United Kingdom, Spain and Italy. Eligible participants were GPs who had been in practice for 3–40 years, spent >70% of time in direct patient care, saw >4 patients with ACS per month, and were personally responsible for prescribing treatment to patients with ACS. The survey included a 20-minute online interview and fieldwork taken place between 18 August to 4 October 2020. All GPs were asked to complete four patient report forms (PRFs).

The patient survey was conducted in six European countries, including France, United Kingdom, Spain, Italy, the Netherlands and Sweden. Eligible participants were patients who had experienced a heart attack in the past 6–18 months. The goal biometrics for the population were 70%/30% split of male/female, 50%/50% split of aged <65 years/≥65 years, ~1:1:1 ratio of 6–9, 10–13, and 14–18 months since ACS event, and a minimum of 50% of patient in each market had only one ACS event. The survey was administered as a 20-minute online interview and fieldwork took place between 27 August and 4 October 2020.

*Ipsos MORI technical note for GP and Patient surveys*

On behalf of Sanofi, Ipsos MORI conducted two separate online surveys:

1) Among 662 patients in seven European countries (France n=143, UK n=150, Spain n=123, Italy n=150, Netherlands n=48 and Sweden n=48) who have experienced a heart attack in the past 6–18 months prior to fieldwork and chose to take part. Fieldwork took place between 27 August and 4 October 2020. Quotas were imposed so the sample consisted of:

- The sample consisted of 446 participants who have experienced one heart attack and 215 who have experienced two or more, one didn’t know the number
- All participants were admitted to hospital for their most recent heart attack
- Across the sample, participants experienced their most recent heart attack 6–9 months prior to the fieldwork (n=263), 10–14 months prior to the fieldwork (n=228) and 15–18 months prior to the fieldwork (n=171)
- A spread of participants across the following age groups with 417 aged 65 and under and 245 aged 65 and over).
- 503 were male, 158 female and one described themselves in another way

2) Among 445 GPs or Primary Care Physicians in four European countries (France n=103, UK n=117, Spain n=117, Italy n=108) and who chose to take part in our research. These participants have been practising for 3–40 years, spend at least 70% of their time in direct patient care (as opposed to research, teaching or administrative duties), see at least five patients with ACS in a typical 3-month period, are personally responsible for discussing and recommending or prescribing medication treatment to patients with ACS and chose to take part. Fieldwork took place between 18 August and 4 October 2020.

## Section 3. Ideation and Hackathon methodology

*Ideation*

The Ideation phase (15–22 April 2021) was conducted using the SkyBoard platform. Participants were invited to generate ideas for each suggested area of improvement by logging onto the platform and adding one digital ‘post-it’ note to each area. The suggested structure for solutions was that they should cover purpose (i.e., what is the solution for), timing (i.e., when to use it), supporting items (i.e., a digital application, printed material), target audience (e.g., nurses, cardiologists, patients) and the expected results. Participants were also able to ‘like’ solutions they found to be the most relevant. In the associated discussion area, participants were able to discuss the suggested proposals (by tagging the person who had made the proposal) and also answer questions themselves.

*Hackathon*

During the hackathon phase (1–9 June 2021), three meetings took place during which hackathon members further co-developed prototype solutions that had been proposed via the ideation platform. Participants had the opportunity to vote for their preferred ideas within the platform. During the third and final hackathon meeting, the hackathon members met to pitch these five solutions; these were presented in PowerPoint format by members of the steering committee. Participants then voted on the most relevant prototypes to be developed. Participants initially voted on the potential replicability and effectiveness of each solution. After reviewing the results of these polls, participants voted again on which solutions should be prioritised for implementation.

**Section 4.** Conditions for receiving PCSK9 inhibitor treatment across selected European countries

| **Country** | **Evolocumab** | **Alirocumab** |
| --- | --- | --- |
| France^1,2^ | Adult patients with ASCVD established by a recent history of MI, non-haemorrhagic stroke and/or symptomatic PAD (secondary prevention) and uncontrolled LDL-C (≥70 mg/dL), in combination with optimised LLT (e.g. comprising at least one statin at the maximum tolerated dose, in combination with ezetimibe) or alone in cases of proven contraindication or intolerance to statins and/or ezetimibe | Adult patients with ASCVD established by a recent history of ACS (secondary prevention) and uncontrolled LDL-C (≥70 mg/dL) despite optimised LLT, in combination with optimised LLT (e.g., comprising at least one statin at the maximum tolerated dose in combination with ezetimibe) |
| Germany^3–6^ | Adult patients with no known ASCVD and mixed dyslipidaemia, not achieving LDL-C goals with the maximum tolerable statin dose, in addition to dietary therapy  Treatment should be in combination with a statin or a statin with other LLTs in patients not achieving LDL-C goals on the maximum tolerable statin dose, or alone/in combination with other LLTs in patients with a statin intolerance/for whom statins are contraindicated | |
| Italy^7^ | Patients aged ≤80 years with mixed dyslipidaemia:   - and LDL-C levels ≥70 mg/dL despite therapy for at least 6 months with high potency statin at maximum tolerated dose + ezetimibe - or after a single LDL-C detection in case of recent AMI (last 12 months) or multiple CV events   or with demonstrated statin intolerance and/or ezetimibe | |
| Netherlands^8^ | For patients with hypercholesterolemia and sufficiently high risk, if a maximum tolerated statin in combination with ezetimibe does not achieve the treatment goal, PCSK9 inhibitor therapy* can be used as follows:   - in combination with both a statin and ezetimibe or: - in combination with ezetimibe alone, in the event of documented statin-intolerance: statin-associated myalgia for at least three different statins, established based on the flow diagram and criteria described by the EAS/ESC consensus   High-risk patients are defined as those who have suffered a CVE and one relapsed CVE, or those who have suffered a CVE and have confirmed statin-intolerance | |
| Spain^9,10^ | Patients with mixed dyslipidaemia and clinical ASCVD in whom intensive LLT with maximum tolerated statin dose and use of other agents (including ezetimibe) has not been sufficient to control LDL-C (once adherence issues and dietary measures have been ruled out), or in cases of statin intolerance  LDL-C threshold > 70, 100 or 130 mg/dL depending upon patient history/risk factors | |

*The source document for this information states Evolocumab in the title, but includes the following statement in the text: ‘Evolocumab is currently included on List 1A, together with the medicinal product alirocumab (Praluent^®^), which also belongs to the class of PCSK9 antibodies. Conditions have been attached to the reimbursement of evolocumab (and of alirocumab), whereby reimbursement is limited to very high-risk groups of patients’.

ACS, acute coronary syndrome; AMI, acute myocardial infarction; ASCVD, atherosclerotic cardiovascular disease; CV, cardiovascular; CVE, cardiovascular event; EAS/ESC, European Atherosclerosis Society/European Society of Cardiology; LDL-C, low density lipoprotein cholesterol; LLT, lipid-lowering therapy; MI, myocardial infarction; PAD, peripheral arterial disease; PCSK9, proprotein convertase subtilisin kexin 9

**References:**

1) <https://www.has-sante.fr/jcms/p_3313603/fr/repatha-evolocumab>

2) <https://www.has-sante.fr/jcms/p_3238105/fr/praluent-alirocumab>

3) <https://www.g-ba.de/downloads/39-261-2517/2016-03-09_AM-RL-XII_Evolocumab_2015-09-15-D-181_BAnz.pdf>

4) <https://www.g-ba.de/downloads/39-261-3468/2018-09-06_AM-RL-XII_Evolocumab_D-345_BAnz.pdf>

5) <https://www.g-ba.de/downloads/39-261-2573/2016-05-04_A;-RL-XII_Alirocumab_2015-11-15-D-194_BAnz.pdf>

6) <https://www.g-ba.de/downloads/39-261-3774/2019-05-02_AM-RL-XII_Alirocumab_D-409_BAnz.pdf>

7) <https://www.aifa.gov.it/-/modifica-registri-di-monitoraggio-repatha-e-praluent-inibitori-di-pcsk-9->

8) <https://english.zorginstituutnederland.nl/publications/reports/2019/01/23/evolocumab-repatha-for-the-treatment-of-primary-hypercholesterolaemia>

9) <https://www.aemps.gob.es/medicamentosUsoHumano/informesPublicos/docs/IPT_13-2020-alirocumab-Praluent.pdf?x10638>

10) <https://www.aemps.gob.es/medicamentosUsoHumano/informesPublicos/docs/IPT_12-2020-evolocumab-Repatha.pdf?x10638>

1. Landmesser U, Pirillo A, Farnier M, et al.: Lipid-lowering therapy and low-density lipoprotein cholesterol goal achievement in patients with acute coronary syndromes: The ACS patient pathway project. *Atheroscler Suppl.* 2020;42:e49–e58.

2. Sionis A, Catapano AL, De Ferrari GM, et al.: Improving lipid management in patients with acute coronary syndrome: The ACS Lipid EuroPath tool. *Atheroscler Suppl.* 2020;42:e65–e71.
